# (4*E*)-*N*-[(2-Chloro­phen­yl)meth­oxy]-1,3-dimethyl-2,6-diphenyl­piperidin-4-imine

**DOI:** 10.1107/S1600536812028899

**Published:** 2012-06-30

**Authors:** Chennan Ramalingan, Seik Weng Ng, Edward R. T. Tiekink

**Affiliations:** a Centre for Nanotechnology, Department of Chemistry, Kalasalingam University, Krishnankoil 626 126, Tamilnadu, India; b Department of Chemistry, University of Malaya, 50603 Kuala Lumpur, Malaysia; c Chemistry Department and Faculty of Science, King Abdulaziz University, PO Box 80203 Jeddah, Saudi Arabia

## Abstract

In the title compound, C_26_H_27_ClN_2_O, the piperidine ring has a chair conformation and all of the ring substituents at C*sp* ^3^ atoms occupy equatorial positions. The dihedral angle formed between the phenyl rings is 48.11 (9)°. The chloro­benzene ring occupies a position orthogonal to the meth­oxy(methyl­idene)amine residue [N—O—C—C torsion angle = −87.90 (15)°]. The conformation about the imine C=N bond [1.278 (2) Å] is *E*, and the chloro substituent is *anti* to the piperidine N atom. Helical supra­molecular chains along [010] are sustained by C—H⋯π inter­actions in the crystal packing.

## Related literature

For the biological activity of mol­ecules having a 2,6-diaryl­piperidine core, see: Ramachandran *et al.* (2011 ▶); Ramalingan *et al.* (2004 ▶). For the structure of the bromo derivative, see: Ramalingan *et al.* (2012 ▶). For the synthesis, see: Ramalingan *et al.* (2006 ▶).
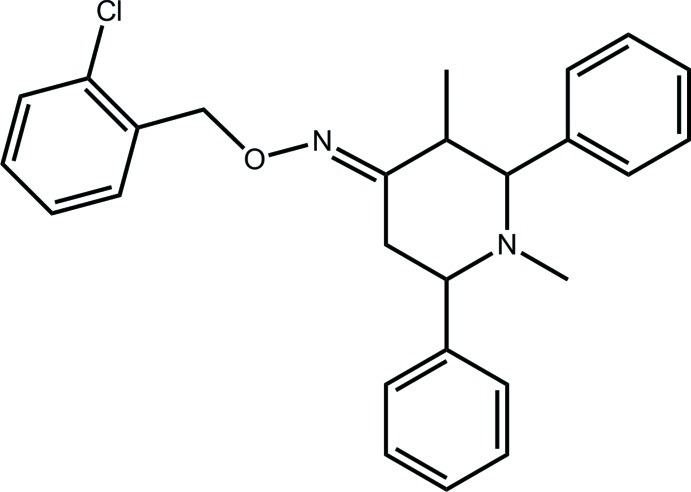


## Experimental

### 

#### Crystal data

C_26_H_27_ClN_2_O
*M* *_r_* = 418.95
Monoclinic, 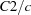

*a* = 20.3043 (8) Å
*b* = 6.8811 (3) Å
*c* = 32.2244 (12) Å
β = 98.478 (4)°
*V* = 4453.1 (3) Å^3^
*Z* = 8
Mo *K*α radiation
μ = 0.19 mm^−1^
*T* = 100 K
0.30 × 0.25 × 0.20 mm

#### Data collection

Agilent SuperNova Dual diffractometer with an Atlas detector
Absorption correction: multi-scan (*CrysAlis PRO*; Agilent, 2012 ▶) *T*_min_ = 0.846, *T*_max_ = 1.000
14305 measured reflections
5108 independent reflections
3847 reflections with *I* > 2σ(*I*)
*R*_int_ = 0.041

#### Refinement

*R*[*F* ^2^ > 2σ(*F* ^2^)] = 0.045
*wR*(*F* ^2^) = 0.118
*S* = 1.02
5108 reflections
271 parameters
H-atom parameters constrained
Δρ_max_ = 0.29 e Å^−3^
Δρ_min_ = −0.28 e Å^−3^

### 

Data collection: *CrysAlis PRO* (Agilent, 2012 ▶); cell refinement: *CrysAlis PRO*; data reduction: *CrysAlis PRO*; program(s) used to solve structure: *SHELXS97* (Sheldrick, 2008 ▶); program(s) used to refine structure: *SHELXL97* (Sheldrick, 2008 ▶); molecular graphics: *ORTEP-3 for Windows* (Farrugia, 1997 ▶) and *DIAMOND* (Brandenburg, 2006 ▶); software used to prepare material for publication: *publCIF* (Westrip, 2010 ▶).

## Supplementary Material

Crystal structure: contains datablock(s) global, I. DOI: 10.1107/S1600536812028899/bt5958sup1.cif

Structure factors: contains datablock(s) I. DOI: 10.1107/S1600536812028899/bt5958Isup2.hkl

Supplementary material file. DOI: 10.1107/S1600536812028899/bt5958Isup3.cml

Additional supplementary materials:  crystallographic information; 3D view; checkCIF report

## Figures and Tables

**Table 1:** Hydrogen-bond geometry (Å, °) *Cg*1 and *Cg*2 are the centroids of the C1–C6 and C15–C20 rings, respectively.

*D*—H⋯*A*	*D*—H	H⋯*A*	*D*⋯*A*	*D*—H⋯*A*
C17—H17⋯*Cg*1^i^	0.95	2.69	3.556 (2)	151
C3—H3⋯*Cg*2^ii^	0.95	2.90	3.6852 (19)	141

Symmetry codes: (i) 
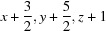
; (ii) 
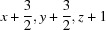
.

**Fig. 1.**
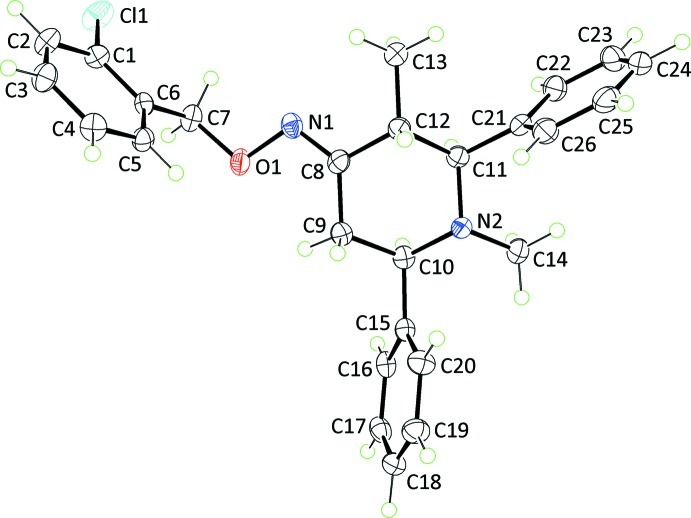
The molecular structure of (I) showing the atom-labelling scheme and displacement ellipsoids at the 50% probability level.

**Fig. 2.**
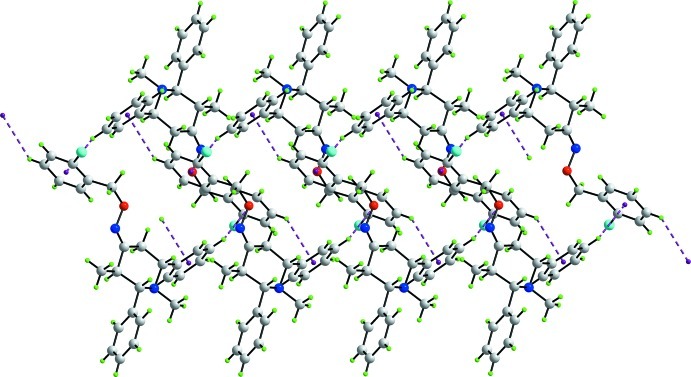
A view of the helical supramolecular chain in (I) sustained by C—H···π interactions which are shown as purple dashed lines.

**Fig. 3.**
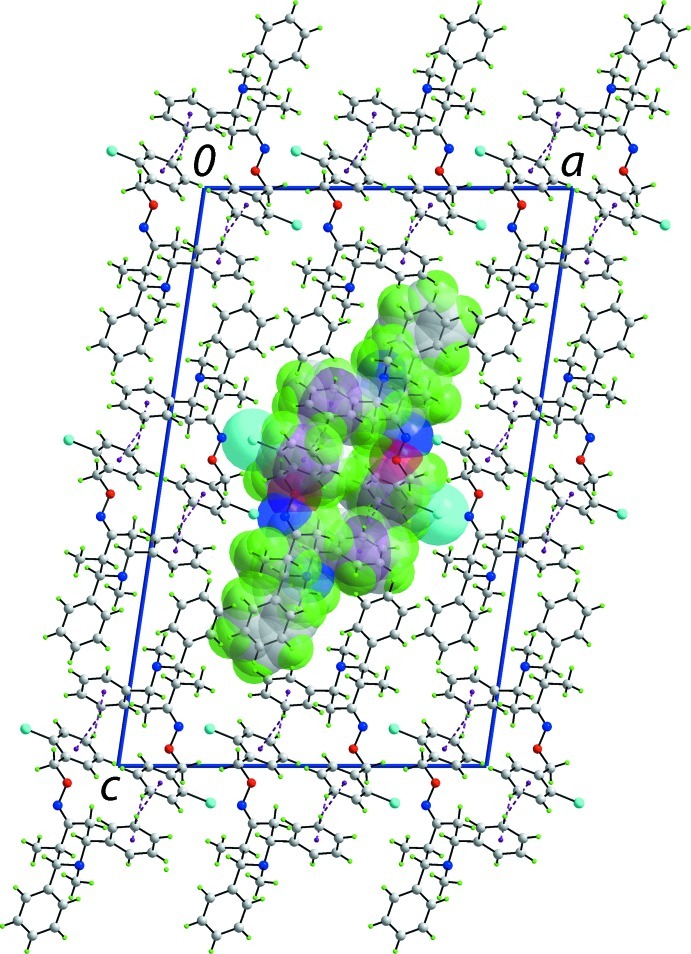
A view in projection down the b axis of the unit-cell contents for (I). The C—H···π interactions are shown as purple dashed lines. One chain has been highlighted in space-filling mode.

## supplementary crystallographic information

### Comment

A diverse range of molecules possessing a 2,6-diarylpiperidine core exhibit potent biological activities (Ramachandran *et al.*, 2011; Ramalingan *et al.*, 2004). In a wide search program towards generating efficient biological agents, the title compound, (I), was synthesized (Ramalingan *et al.*, 2006). Herein, the crystal and molecular structure of (I) is described.

In (I), Fig. 1, the piperidine ring has a chair conformation and all of the ring-substituents occupy equatorial positions. In the bromo derivative (Ramalingan *et al.*, 2012) all but the *N*-bound substituent, which occupies a bisectional position, also occupy equatorial positions, in accord with (I). The dihedral angle formed between the C15–C20 and C21–C26 phenyl rings is 48.11 (9)°, and each forms a dihedral angle of 64.68 (8) and 72.57 (9)°, respectively, with the chlorobenzene ring, which occupies a position orthogonal to the methoxy(methylidene)amine residue as seen in the N1—O1—C7—C6 torsion angle of -87.90 (15)°. The conformation about the imine C8═N1 bond [1.278 (2) Å] is *E*. The chloro substituent is *anti* to the piperidine-N atom.

In the crystal packing, helical supramolecular chains along [0 1 0] are sustained by C—H···π interactions, Fig. 2 and Table 1. These assemble into a three-dimensional architecture without specific intermolecular interactions between the chains, Fig. 3.

### Experimental

For full details of the synthesis, refer to Ramalingan *et al.* (2006). Re-crystallization was performed by slow evaporation of an ethanolic solution of (I) which afforded colourless crystals. *M*.pt: 361–362 K.

### Refinement

Carbon-bound H-atoms were placed in calculated positions [C—H = 0.95–0.99 Å, *U*_iso_(H) = 1.2–1.5*U*_eq_(C)] and were included in the refinement in the riding model approximation. Owing to poor agreement, two reflections, *i.e*. (0 1 0) and (1 2 10), were omitted from the final refinement.

### Crystal data

**Table:**

C_26_H_27_ClN_2_O	*F*(000) = 1776
*M**_r_* = 418.95	*D*_x_ = 1.250 Mg m^−^^3^
Monoclinic, *C*2/*c*	Mo *K*α radiation, λ = 0.71073 Å
Hall symbol: -C 2yc	Cell parameters from 4543 reflections
*a* = 20.3043 (8) Å	θ = 2.2–27.5°
*b* = 6.8811 (3) Å	µ = 0.19 mm^−^^1^
*c* = 32.2244 (12) Å	*T* = 100 K
β = 98.478 (4)°	Prism, colourless
*V* = 4453.1 (3) Å^3^	0.30 × 0.25 × 0.20 mm
*Z* = 8	

### Data collection

**Table:**

Agilent SuperNova Dual diffractometer with an Atlas detector	5108 independent reflections
Radiation source: SuperNova (Mo) X-ray Source	3847 reflections with *I* > 2σ(*I*)
Mirror monochromator	*R*_int_ = 0.041
Detector resolution: 10.4041 pixels mm^-1^	θ_max_ = 27.6°, θ_min_ = 2.2°
ω scan	*h* = −19→26
Absorption correction: multi-scan (*CrysAlis PRO*; Agilent, 2012)	*k* = −8→8
*T*_min_ = 0.846, *T*_max_ = 1.000	*l* = −41→40
14305 measured reflections	

### Refinement

**Table:**

Refinement on *F*^2^	Primary atom site location: structure-invariant direct methods
Least-squares matrix: full	Secondary atom site location: difference Fourier map
*R*[*F*^2^ > 2σ(*F*^2^)] = 0.045	Hydrogen site location: inferred from neighbouring sites
*wR*(*F*^2^) = 0.118	H-atom parameters constrained
*S* = 1.02	*w* = 1/[σ^2^(*F*_o_^2^) + (0.0478*P*)^2^ + 2.1668*P*] where *P* = (*F*_o_^2^ + 2*F*_c_^2^)/3
5108 reflections	(Δ/σ)_max_ = 0.001
271 parameters	Δρ_max_ = 0.29 e Å^−^^3^
0 restraints	Δρ_min_ = −0.28 e Å^−^^3^

### Special details

**Table:**

Geometry. All e.s.d.'s (except the e.s.d. in the dihedral angle between two l.s. planes) are estimated using the full covariance matrix. The cell e.s.d.'s are taken into account individually in the estimation of e.s.d.'s in distances, angles and torsion angles; correlations between e.s.d.'s in cell parameters are only used when they are defined by crystal symmetry. An approximate (isotropic) treatment of cell e.s.d.'s is used for estimating e.s.d.'s involving l.s. planes.
Refinement. Refinement of *F*^2^ against ALL reflections. The weighted *R*-factor *wR* and goodness of fit *S* are based on *F*^2^, conventional *R*-factors *R* are based on *F*, with *F* set to zero for negative *F*^2^. The threshold expression of *F*^2^ > σ(*F*^2^) is used only for calculating *R*-factors(gt) *etc*. and is not relevant to the choice of reflections for refinement. *R*-factors based on *F*^2^ are statistically about twice as large as those based on *F*, and *R*- factors based on ALL data will be even larger.

### Fractional atomic coordinates and isotropic or equivalent isotropic displacement parameters (Å^2^)

**Table:**

	*x*	*y*	*z*	*U*_iso_*/*U*_eq_	
Cl1	0.76604 (2)	0.38812 (7)	0.564062 (15)	0.03838 (15)	
O1	0.63144 (6)	0.72592 (17)	0.47058 (3)	0.0276 (3)	
N1	0.64825 (7)	0.6556 (2)	0.43184 (4)	0.0244 (3)	
N2	0.56479 (6)	0.9658 (2)	0.32797 (4)	0.0210 (3)	
C1	0.68498 (8)	0.3178 (2)	0.54424 (5)	0.0233 (4)	
C2	0.66062 (9)	0.1447 (3)	0.55791 (5)	0.0287 (4)	
H2	0.6880	0.0643	0.5773	0.034*	
C3	0.59581 (9)	0.0907 (3)	0.54287 (5)	0.0280 (4)	
H3	0.5783	−0.0271	0.5521	0.034*	
C4	0.55655 (8)	0.2081 (3)	0.51448 (5)	0.0258 (4)	
H4	0.5122	0.1705	0.5041	0.031*	
C5	0.58196 (8)	0.3814 (2)	0.50108 (5)	0.0221 (4)	
H5	0.5546	0.4610	0.4815	0.027*	
C6	0.64648 (8)	0.4393 (2)	0.51585 (5)	0.0199 (3)	
C7	0.67419 (9)	0.6302 (3)	0.50347 (5)	0.0256 (4)	
H7A	0.6815	0.7164	0.5283	0.031*	
H7B	0.7179	0.6071	0.4943	0.031*	
C8	0.61070 (8)	0.7273 (2)	0.40023 (5)	0.0227 (4)	
C9	0.55388 (8)	0.8646 (3)	0.40074 (5)	0.0260 (4)	
H9A	0.5550	0.9163	0.4295	0.031*	
H9B	0.5115	0.7929	0.3932	0.031*	
C10	0.55575 (8)	1.0353 (2)	0.37012 (5)	0.0219 (4)	
H10	0.5940	1.1217	0.3810	0.026*	
C11	0.62664 (8)	0.8506 (2)	0.33000 (5)	0.0214 (4)	
H11	0.6646	0.9332	0.3431	0.026*	
C12	0.62416 (8)	0.6675 (2)	0.35738 (5)	0.0223 (4)	
H12	0.5858	0.5863	0.3443	0.027*	
C13	0.68726 (9)	0.5454 (3)	0.35879 (5)	0.0286 (4)	
H13A	0.6845	0.4323	0.3769	0.043*	
H13B	0.7261	0.6240	0.3700	0.043*	
H13C	0.6917	0.5016	0.3304	0.043*	
C14	0.56727 (9)	1.1334 (3)	0.30007 (5)	0.0288 (4)	
H14A	0.5749	1.0881	0.2723	0.043*	
H14B	0.6037	1.2199	0.3118	0.043*	
H14C	0.5250	1.2040	0.2975	0.043*	
C15	0.49163 (8)	1.1488 (2)	0.36936 (5)	0.0213 (3)	
C16	0.49022 (8)	1.3168 (2)	0.39308 (5)	0.0237 (4)	
H16	0.5306	1.3691	0.4074	0.028*	
C17	0.43008 (9)	1.4094 (3)	0.39607 (5)	0.0271 (4)	
H17	0.4296	1.5236	0.4126	0.033*	
C18	0.37128 (9)	1.3360 (3)	0.37519 (5)	0.0277 (4)	
H18	0.3302	1.3975	0.3778	0.033*	
C19	0.37231 (9)	1.1719 (3)	0.35031 (5)	0.0295 (4)	
H19	0.3320	1.1231	0.3352	0.035*	
C20	0.43214 (8)	1.0787 (3)	0.34742 (5)	0.0269 (4)	
H20	0.4325	0.9663	0.3304	0.032*	
C21	0.63939 (8)	0.7940 (2)	0.28645 (5)	0.0220 (4)	
C22	0.69737 (9)	0.8528 (3)	0.27206 (5)	0.0283 (4)	
H22	0.7291	0.9288	0.2897	0.034*	
C23	0.70963 (10)	0.8021 (3)	0.23233 (6)	0.0354 (5)	
H23	0.7494	0.8442	0.2228	0.042*	
C24	0.66437 (10)	0.6911 (3)	0.20661 (6)	0.0346 (5)	
H24	0.6727	0.6571	0.1793	0.042*	
C25	0.60686 (10)	0.6291 (3)	0.22063 (6)	0.0340 (4)	
H25	0.5758	0.5511	0.2030	0.041*	
C26	0.59421 (9)	0.6802 (3)	0.26037 (5)	0.0289 (4)	
H26	0.5545	0.6373	0.2698	0.035*	

### Atomic displacement parameters (Å^2^)

**Table:**

	*U*^11^	*U*^22^	*U*^33^	*U*^12^	*U*^13^	*U*^23^
Cl1	0.0221 (2)	0.0391 (3)	0.0494 (3)	−0.00554 (19)	−0.00987 (19)	0.0090 (2)
O1	0.0368 (7)	0.0258 (7)	0.0208 (6)	0.0004 (5)	0.0067 (5)	0.0050 (5)
N1	0.0284 (8)	0.0227 (8)	0.0231 (7)	−0.0036 (6)	0.0070 (6)	0.0011 (6)
N2	0.0207 (7)	0.0217 (7)	0.0205 (7)	0.0014 (6)	0.0030 (5)	0.0023 (6)
C1	0.0201 (8)	0.0235 (9)	0.0254 (8)	−0.0021 (7)	0.0002 (6)	−0.0009 (7)
C2	0.0303 (10)	0.0243 (10)	0.0293 (9)	0.0004 (7)	−0.0023 (7)	0.0061 (8)
C3	0.0324 (10)	0.0208 (9)	0.0302 (9)	−0.0057 (7)	0.0029 (7)	0.0031 (8)
C4	0.0230 (9)	0.0264 (9)	0.0271 (9)	−0.0069 (7)	0.0005 (7)	−0.0020 (8)
C5	0.0224 (9)	0.0249 (9)	0.0182 (8)	−0.0005 (7)	0.0002 (6)	0.0003 (7)
C6	0.0230 (8)	0.0215 (9)	0.0154 (7)	−0.0013 (6)	0.0042 (6)	−0.0005 (7)
C7	0.0275 (9)	0.0259 (10)	0.0222 (8)	−0.0054 (7)	0.0000 (7)	0.0034 (7)
C8	0.0211 (9)	0.0205 (9)	0.0264 (9)	−0.0034 (7)	0.0036 (7)	0.0053 (7)
C9	0.0264 (9)	0.0273 (10)	0.0256 (9)	0.0020 (7)	0.0088 (7)	0.0045 (7)
C10	0.0217 (9)	0.0226 (9)	0.0211 (8)	−0.0029 (7)	0.0017 (6)	−0.0015 (7)
C11	0.0178 (8)	0.0231 (9)	0.0230 (8)	−0.0016 (6)	0.0020 (6)	0.0004 (7)
C12	0.0191 (8)	0.0241 (9)	0.0238 (8)	−0.0010 (7)	0.0033 (6)	0.0025 (7)
C13	0.0287 (10)	0.0310 (10)	0.0266 (9)	0.0065 (8)	0.0058 (7)	0.0053 (8)
C14	0.0345 (10)	0.0281 (10)	0.0244 (9)	0.0041 (8)	0.0057 (7)	0.0051 (8)
C15	0.0237 (9)	0.0211 (9)	0.0194 (8)	−0.0012 (7)	0.0041 (6)	0.0016 (7)
C16	0.0292 (9)	0.0235 (9)	0.0187 (8)	−0.0060 (7)	0.0047 (7)	−0.0001 (7)
C17	0.0384 (11)	0.0199 (9)	0.0255 (9)	0.0007 (7)	0.0129 (7)	−0.0006 (7)
C18	0.0287 (10)	0.0285 (10)	0.0275 (9)	0.0063 (7)	0.0092 (7)	0.0026 (8)
C19	0.0229 (9)	0.0339 (10)	0.0309 (9)	−0.0002 (7)	0.0008 (7)	−0.0027 (8)
C20	0.0251 (9)	0.0260 (9)	0.0295 (9)	−0.0011 (7)	0.0031 (7)	−0.0078 (8)
C21	0.0223 (9)	0.0219 (9)	0.0213 (8)	0.0037 (7)	0.0018 (6)	0.0038 (7)
C22	0.0309 (10)	0.0261 (10)	0.0286 (9)	−0.0006 (7)	0.0069 (7)	0.0043 (8)
C23	0.0391 (11)	0.0359 (11)	0.0347 (10)	0.0077 (9)	0.0173 (8)	0.0091 (9)
C24	0.0441 (12)	0.0369 (11)	0.0234 (9)	0.0187 (9)	0.0069 (8)	0.0024 (8)
C25	0.0356 (11)	0.0360 (11)	0.0273 (9)	0.0094 (8)	−0.0058 (8)	−0.0036 (8)
C26	0.0233 (9)	0.0362 (11)	0.0261 (9)	0.0011 (8)	−0.0004 (7)	−0.0018 (8)

### Geometric parameters (Å, º)

**Table:**

Cl1—C1	1.7439 (17)	C12—C13	1.527 (2)
O1—N1	1.4266 (17)	C12—H12	1.0000
O1—C7	1.4280 (19)	C13—H13A	0.9800
N1—C8	1.278 (2)	C13—H13B	0.9800
N2—C14	1.467 (2)	C13—H13C	0.9800
N2—C11	1.479 (2)	C14—H14A	0.9800
N2—C10	1.477 (2)	C14—H14B	0.9800
C1—C2	1.386 (2)	C14—H14C	0.9800
C1—C6	1.391 (2)	C15—C16	1.389 (2)
C2—C3	1.384 (2)	C15—C20	1.393 (2)
C2—H2	0.9500	C16—C17	1.393 (2)
C3—C4	1.382 (2)	C16—H16	0.9500
C3—H3	0.9500	C17—C18	1.377 (3)
C4—C5	1.393 (2)	C17—H17	0.9500
C4—H4	0.9500	C18—C19	1.387 (3)
C5—C6	1.385 (2)	C18—H18	0.9500
C5—H5	0.9500	C19—C20	1.389 (2)
C6—C7	1.506 (2)	C19—H19	0.9500
C7—H7A	0.9900	C20—H20	0.9500
C7—H7B	0.9900	C21—C26	1.391 (2)
C8—C9	1.493 (2)	C21—C22	1.388 (2)
C8—C12	1.504 (2)	C22—C23	1.385 (2)
C9—C10	1.538 (2)	C22—H22	0.9500
C9—H9A	0.9900	C23—C24	1.375 (3)
C9—H9B	0.9900	C23—H23	0.9500
C10—C15	1.515 (2)	C24—C25	1.380 (3)
C10—H10	1.0000	C24—H24	0.9500
C11—C21	1.515 (2)	C25—C26	1.388 (2)
C11—C12	1.543 (2)	C25—H25	0.9500
C11—H11	1.0000	C26—H26	0.9500
			
N1—O1—C7	107.18 (12)	C13—C12—C11	111.60 (13)
C8—N1—O1	112.01 (13)	C8—C12—H12	107.7
C14—N2—C11	110.05 (12)	C13—C12—H12	107.7
C14—N2—C10	109.23 (13)	C11—C12—H12	107.7
C11—N2—C10	110.66 (12)	C12—C13—H13A	109.5
C2—C1—C6	122.22 (15)	C12—C13—H13B	109.5
C2—C1—Cl1	118.92 (13)	H13A—C13—H13B	109.5
C6—C1—Cl1	118.84 (13)	C12—C13—H13C	109.5
C3—C2—C1	118.97 (16)	H13A—C13—H13C	109.5
C3—C2—H2	120.5	H13B—C13—H13C	109.5
C1—C2—H2	120.5	N2—C14—H14A	109.5
C4—C3—C2	120.05 (16)	N2—C14—H14B	109.5
C4—C3—H3	120.0	H14A—C14—H14B	109.5
C2—C3—H3	120.0	N2—C14—H14C	109.5
C3—C4—C5	120.12 (16)	H14A—C14—H14C	109.5
C3—C4—H4	119.9	H14B—C14—H14C	109.5
C5—C4—H4	119.9	C16—C15—C20	118.76 (16)
C6—C5—C4	120.97 (15)	C16—C15—C10	120.51 (15)
C6—C5—H5	119.5	C20—C15—C10	120.56 (15)
C4—C5—H5	119.5	C15—C16—C17	120.54 (16)
C5—C6—C1	117.67 (15)	C15—C16—H16	119.7
C5—C6—C7	122.10 (15)	C17—C16—H16	119.7
C1—C6—C7	120.18 (14)	C18—C17—C16	120.28 (16)
O1—C7—C6	112.89 (13)	C18—C17—H17	119.9
O1—C7—H7A	109.0	C16—C17—H17	119.9
C6—C7—H7A	109.0	C17—C18—C19	119.67 (16)
O1—C7—H7B	109.0	C17—C18—H18	120.2
C6—C7—H7B	109.0	C19—C18—H18	120.2
H7A—C7—H7B	107.8	C18—C19—C20	120.18 (16)
N1—C8—C9	127.36 (15)	C18—C19—H19	119.9
N1—C8—C12	117.26 (15)	C20—C19—H19	119.9
C9—C8—C12	115.38 (14)	C19—C20—C15	120.51 (16)
C8—C9—C10	112.57 (13)	C19—C20—H20	119.7
C8—C9—H9A	109.1	C15—C20—H20	119.7
C10—C9—H9A	109.1	C26—C21—C22	118.62 (15)
C8—C9—H9B	109.1	C26—C21—C11	121.13 (15)
C10—C9—H9B	109.1	C22—C21—C11	120.25 (15)
H9A—C9—H9B	107.8	C23—C22—C21	120.83 (18)
N2—C10—C15	112.02 (13)	C23—C22—H22	119.6
N2—C10—C9	111.17 (13)	C21—C22—H22	119.6
C15—C10—C9	107.56 (13)	C24—C23—C22	120.14 (18)
N2—C10—H10	108.7	C24—C23—H23	119.9
C15—C10—H10	108.7	C22—C23—H23	119.9
C9—C10—H10	108.7	C23—C24—C25	119.79 (17)
N2—C11—C21	110.91 (13)	C23—C24—H24	120.1
N2—C11—C12	111.35 (12)	C25—C24—H24	120.1
C21—C11—C12	110.13 (13)	C24—C25—C26	120.29 (18)
N2—C11—H11	108.1	C24—C25—H25	119.9
C21—C11—H11	108.1	C26—C25—H25	119.9
C12—C11—H11	108.1	C21—C26—C25	120.32 (17)
C8—C12—C13	112.86 (14)	C21—C26—H26	119.8
C8—C12—C11	109.08 (14)	C25—C26—H26	119.8
			
C7—O1—N1—C8	177.41 (14)	N1—C8—C12—C11	−131.39 (16)
C6—C1—C2—C3	0.0 (3)	C9—C8—C12—C11	49.02 (18)
Cl1—C1—C2—C3	178.66 (13)	N2—C11—C12—C8	−55.90 (17)
C1—C2—C3—C4	0.4 (3)	C21—C11—C12—C8	−179.37 (13)
C2—C3—C4—C5	−0.3 (3)	N2—C11—C12—C13	178.71 (13)
C3—C4—C5—C6	−0.1 (2)	C21—C11—C12—C13	55.24 (18)
C4—C5—C6—C1	0.4 (2)	N2—C10—C15—C16	−138.80 (15)
C4—C5—C6—C7	−177.07 (15)	C9—C10—C15—C16	98.76 (17)
C2—C1—C6—C5	−0.3 (2)	N2—C10—C15—C20	46.1 (2)
Cl1—C1—C6—C5	−179.05 (12)	C9—C10—C15—C20	−76.34 (18)
C2—C1—C6—C7	177.20 (16)	C20—C15—C16—C17	2.3 (2)
Cl1—C1—C6—C7	−1.5 (2)	C10—C15—C16—C17	−172.88 (14)
N1—O1—C7—C6	−87.90 (15)	C15—C16—C17—C18	−0.6 (2)
C5—C6—C7—O1	−9.4 (2)	C16—C17—C18—C19	−1.5 (3)
C1—C6—C7—O1	173.12 (14)	C17—C18—C19—C20	1.9 (3)
O1—N1—C8—C9	−1.6 (2)	C18—C19—C20—C15	−0.1 (3)
O1—N1—C8—C12	178.88 (13)	C16—C15—C20—C19	−2.0 (2)
N1—C8—C9—C10	133.48 (17)	C10—C15—C20—C19	173.19 (15)
C12—C8—C9—C10	−47.0 (2)	N2—C11—C21—C26	−60.8 (2)
C14—N2—C10—C15	60.47 (17)	C12—C11—C21—C26	62.9 (2)
C11—N2—C10—C15	−178.23 (13)	N2—C11—C21—C22	120.20 (16)
C14—N2—C10—C9	−179.16 (13)	C12—C11—C21—C22	−116.07 (17)
C11—N2—C10—C9	−57.86 (17)	C26—C21—C22—C23	1.0 (3)
C8—C9—C10—N2	49.92 (19)	C11—C21—C22—C23	−179.98 (16)
C8—C9—C10—C15	172.88 (14)	C21—C22—C23—C24	−0.4 (3)
C14—N2—C11—C21	−54.15 (17)	C22—C23—C24—C25	−0.4 (3)
C10—N2—C11—C21	−174.97 (13)	C23—C24—C25—C26	0.7 (3)
C14—N2—C11—C12	−177.17 (13)	C22—C21—C26—C25	−0.7 (3)
C10—N2—C11—C12	62.01 (17)	C11—C21—C26—C25	−179.72 (16)
N1—C8—C12—C13	−6.7 (2)	C24—C25—C26—C21	−0.1 (3)
C9—C8—C12—C13	173.67 (14)		

### Hydrogen-bond geometry (Å, º)

Cg1 and Cg2 are the centroids of the C1–C6 and C15–C20 rings, respectively.

**Table:**

*D*—H···*A*	*D*—H	H···*A*	*D*···*A*	*D*—H···*A*
C17—H17···*Cg*1^i^	0.95	2.69	3.556 (2)	151
C3—H3···*Cg*2^ii^	0.95	2.90	3.6852 (19)	141

Symmetry codes: (i) *x*+3/2, *y*+5/2, *z*+1; (ii) *x*+3/2, *y*+3/2, *z*+1.
